# Metabolomics Insights into Gut Microbiota and Functional Constipation

**DOI:** 10.3390/metabo15040269

**Published:** 2025-04-12

**Authors:** Fan Zheng, Yong Yang, Guanting Lu, Joo Shun Tan, Uma Mageswary, Yu Zhan, Mina Ehab Ayad, Yeong-Yeh Lee, Daoyuan Xie

**Affiliations:** 1 Deyang People’s Hospital of Chengdu University of Traditional Chinese Medicine, Deyang 617000, China; fanzheng123@student.usm.my (F.Z.); yongyz88@126.com (Y.Y.); guantlv@126.com (G.L.); 2 School of Medical Sciences, University Sains Malaysia, Kota Bharu 16150, Malaysia; mina.ehab.td3@med.bsu.edu.eg; 3 School of Industrial Technology, University Sains Malaysia, Penang 11700, Malaysia; jooshun@usm.my (J.S.T.); umamageswary@student.usm.my (U.M.); 4 Anorectal Department, Chengdu Integrated TCM & Western Medicine Hospital, Chengdu 610000, China; zhanyu@cdutcm.edu.cn; 5 GI Function and Motility Unit, Hospital Pakar University Sains Malaysia, Kota Bharu 16150, Malaysia

**Keywords:** metabolomics, gut microbiota, functional constipation, short-chain fatty acids, personalization

## Abstract

**Background:** The composition and metabolic activity of the gut microbiota play a crucial role in various health conditions, including the occurrence and development of chronic constipation. Recent metabolomic advances reveal that gut microbiota-derived metabolites—such as SCFAs, bile acids, neurotransmitters, and microbial gases—play critical roles in regulating intestinal function. **Methods:** We systematically analyzed the current literature on microbial metabolomics in chronic constipation. This review consolidates findings from high-throughput metabolomic techniques (GC-MS, LC-MS, NMR) comparing metabolic profiles of constipated patients with healthy individuals. It also examines diagnostic improvements and personalized treatments, including fecal microbiota transplantation and neuromodulation, guided by these metabolomic insights. **Results:** This review shows that reduced SCFA levels impair intestinal motility and promote inflammation. An altered bile acid metabolism—with decreased secondary bile acids like deoxycholic acid—disrupts receptor-mediated signaling, further affecting motility. Additionally, imbalances in amino acid metabolism and neurotransmitter production contribute to neuromuscular dysfunction, while variations in microbial gas production (e.g., methane vs. hydrogen) further modulate gut transit. **Conclusions:** Integrating metabolomics with gut microbiota research clarifies how specific microbial metabolites regulate gut function. These insights offer promising directions for precision diagnostics and targeted therapies to restore microbial balance and improve intestinal motility.

## 1. Introduction

Constipation has emerged as a significant global health concern, exerting detrimental impacts not only on physical wellbeing but also contributing to psychological distress and imposing substantial socioeconomic burdens [1]. The characteristics of constipation are unsatisfactory bowel movements, mainly including difficulty in passing stools, dry stools, and a reduced frequency of bowel movements [2]. Research shows that the occurrence of constipation is related to many factors, including lifestyle, education level, social class, and other social factors, as well as physiological factors such as gender and age [3,4]. A meta-analysis that included 26 studies showed that the pooled prevalence of chronic constipation in women is nearly twice that of men [5]. The prevalence of chronic constipation increases with age [6]. Approximately 26% of men and 34% of women aged 65 and older experience symptoms of constipation [7,8].

This multifactorial disorder demonstrates complex systemic associations, extending beyond its primary gastrointestinal manifestations to influence multiple organ systems. Pathophysiological studies reveal that chronic constipation is intimately associated with proctological conditions including hemorrhoids, anal fissures, and rectal prolapse [9]. More importantly, emerging evidence positions constipation as a potential modulator in the pathogenesis of various systemic diseases, notably colorectal carcinoma [10], hepatic encephalopathy [11], mammary gland pathologies [12], and neurodegenerative disorders such as Alzheimer’s disease [13]. Recent meta-analyses have established a significant correlation between prolonged constipation and elevated risks of cardiovascular events, particularly venous thromboembolism (VTE), with mechanistic studies suggesting platelet activation mediated by the gut–brain axis as a plausible pathway [14]. The pathophysiology of chronic constipation is complex and can be classified into primary constipation and secondary constipation based on the etiology [1,15]. Unlike primary constipation which originates from intrinsic intestinal dysregulation, secondary constipation typically arises as a concomitant manifestation of underlying systemic pathologies or iatrogenic interventions. Its etiology mainly includes organic diseases (such as colon tumors or colonic strictures), medication use (such as opioids, anticholinergic drugs, calcium channel blockers), or underlying diseases (such as metabolic or neurogenic disorders), while primary constipation is a neuromuscular dysfunction of the sensory and motor functions of the colon or rectum [15,16].

Despite the high prevalence of constipation, its pathophysiology remains incompletely understood. Recent years have witnessed rapid advancements in metabolomics, providing novel perspectives for investigating the intricate interactions between gut microbiota and host health/disease [17]. Research shows that the gut microbiome of patients with constipation differs significantly from that of the normal population. Bifidobacteria and lactobacilli are usually less prevalent in patients with constipation [18]. By analyzing dynamic alterations in endogenous metabolites, metabolomics has unveiled the pivotal metabolic roles played by gut microbiota in constipated individuals. Research demonstrates that gut microbiota not only plays a pivotal role in maintaining normal physiological functions but also exhibits significant associations with various pathological conditions, particularly the role of gut microbiota in its development and progression [19]. Therefore, the application of metabolomics-driven approaches enables the systematic identification of novel therapeutic targets for chronic constipation management, specifically through the modulation of gut microbiome–host metabolic crosstalk [20]. Emerging evidence suggests that precision interventions targeting microbial-derived metabolites, such as secondary bile acids (BAs) and short-chain fatty acids (SCFAs), not only ameliorate core symptomatology, but also restore systemic metabolic homeostasis via enterohepatic circuity regulation. Crucially, these pathophysiology-informed strategies demonstrate favorable safety profiles with negligible iatrogenic risks compared to conventional prokinetic agents. The development of such mechanism-informed therapeutic strategies might therefore represent a paradigm shift in functional gastrointestinal disorder management, offering sustainable clinical solutions for refractory patients.

Metabolomic studies have revealed that the metabolite profiles in patients with functional constipation (FC) often show significant differences compared to healthy individuals, particularly in the production of specific metabolites. Dysbiosis of the gut microbiome may lead to a decrease in SCFAs levels, which can trigger gastrointestinal dysfunctions such as constipation [21]. Therefore, regulating the gut microbiome to promote the production of SCFAs may be an effective strategy for improving gut health and alleviating constipation symptoms [22]. In addition to the significant effects of SCFAs on gut function, inflammatory response, and the immune system, the metabolic characteristics of patients with constipation also include abnormal amino acid metabolism, changes in lipid metabolism, and alterations in gut microbial metabolites. In terms of amino acid metabolism abnormalities, research has found that certain amino acids in patients with constipation, such as tryptophan and glycine, have significantly altered levels of metabolic products, which may be related to intestinal motility [23]. For instance, upstream products related to arginine biosynthesis in the serum of FC patients, such as 2-oxoglutarate, L-glutamate, N-acetylornithine, and L-ornithine, are significantly reduced, which may serve as potential diagnostic markers for FC [19]. In addition, the abundance of Bacteroides and certain butyrate-producing bacteria (such as *Roseburia*, *Faecaliberium*, and *Butyriccoccus*) in the intestines of FC patients increases, indicating that these microorganisms may play an important role in the occurrence of constipation [19], especially SCFAs such as butyrate and propionate. These metabolites not only act on G protein-coupled receptors (such as GPR41 and GPR43) to promote intestinal motility but also enhance gut barrier function and regulate immune responses [24]. The fatty acid profile of constipated mice is abnormal, mainly characterized by a decrease in free fatty acids and an increase in certain long-chain fatty acids, which may be related to changes in their gut microbiota [25]. These changes indicate that the dysbiosis of the gut microbiota directly affects the occurrence of constipation. These findings emphasize the key role of microbial metabolites in gut health and provide a theoretical basis for microbiome-based therapeutic approaches.

Based on the latest trends in gut microbiome research, there are new insights and strategies for the diagnosis, personalized treatment, and health management of constipation.

### 1.1. Diagnostic Advancements

Recent diagnostic advancements integrate microbial profiling with a functional motility assessment to refine constipation subtyping. Akkermansia muciniphila abundance ≤0.8% of total microbiota and butyrate concentration <12 μmol/g stool effectively discriminate slow-transit constipation [26,27]. For neurogastrodynamic evaluation, the wireless motility capsule (WMC) demonstrates unique diagnostic value in neurogenic constipation, particularly for idiopathic autonomic neuropathy-related cases. In a 20-patient cohort, WMC identified foregut/midgut transit delays (50%) and severe hindgut contractility deficits (85%), correlating strongly with COMPASS-31 gastrointestinal symptom scores. Its quantitative motility parameters showed neuroanatomical alignment with autonomic dysfunction markers (cardiovagal/sudomotor testing), enabling the objective mapping of enteric nervous system impairment. These findings establish WMC as a novel tool for localizing segment-specific dysmotility patterns and unraveling autonomic-enteric pathophysiological interactions in constipation diagnostics [28,29].

### 1.2. Personalized Therapeutic Interventions

One of the personalized therapeutic interventions is microbiota-directed therapy. Bifidobacterium longum BB536 shows improvement in weekly spontaneous bowel movements versus placebo (*p* < 0.01) in methane-positive constipation [30,31,32]. Fecal microbiota transplantation (FMT) from high-butyrate producers (>18 μmol/g) demonstrates superior efficacy in restoring motility [33]. Another is brain–gut axis modulation. In a 4-week randomized trial with 40 IBS-C patients, taVNS outperformed the sham stimulation by significantly improving pain (VAS), bowel habits, psychological scores, and quality of life (*p* < 0.001), while enhancing vagal activity and modulating serum acetylcholine/nitric oxide (NO) levels. The intervention concurrently increased Bifidobacterium abundance and short-chain fatty acid production (acetate/butyrate/propionate) but reduced tryptophan metabolites, with favorable safety profiles (3 vs. 2 adverse events) [34].

### 1.3. Health Management of Constipation

Recent advances in constipation management demonstrate multi-modal breakthroughs: It is certain that a personalized dietary fiber diet can improve the perception of bowel activity and exert positive changes in individuals with functional constipation [35,36]. Methods of neuromodulation used to treat constipation mainly choose the direct stimulation of sacral nerves, which is called sacral neuromodulation (SNM) [37]. One clinical study evaluated a two-stage neuromodulation protocol in 21 refractory constipation patients (95% female). The intervention demonstrated a 57% acute therapeutic response (12/21, *p* < 0.01) during the temporary stimulation phase, with 11 proceeding to permanent implantation showing sustained efficacy at 38-month median follow-up (IQR 18–62 months), albeit 27% (3/11) required surgical revisions for electrode migration or pulse generator malfunctions. The evidence supports sacral neuromodulation as a viable second-line therapy for rigorously screened patients (Oxford CEBM Level 2b) [38]. Also, currently, some products like AI-powered toilet platforms also participate in research such as integrating multi-omics analytics.

## 2. The Relationship Between Gut Microbiota and Constipation

### 2.1. Normal Microbiota and Constipation Balance

Healthy human guts contain microorganisms including bacteria, fungi, and viruses. According to taxonomy, the gut microbiota can be divided into multiple levels, including phylum, class, order, family, and genus. Recent data have updated the number of bacteria inhabiting the human body to 381,013, a number of the same order of human cells [39].

A healthy gut microbiome is essential for regulating immune responses, reducing chronic inflammation, and lowering the risk of gastrointestinal diseases [40]. By regulating intestinal barrier integrity, enhancing intestinal motility, and sustaining optimal luminal pH levels, it actively prevents constipation and mitigates risks of gastrointestinal disorders. The gut microbiota influences both nutrient absorption and bowel movement regulation. It helps prevent constipation and gastrointestinal diseases by regulating the gut barrier function, promoting intestinal motility, and balancing gut pH levels. Research shows that normal microbiota can enhance gut movement and increase intestinal contractions by producing SCFAs to improve the frequency and quality of bowel movements [41]. A healthy gut contains diverse beneficial bacteria that produce SCFAs like butyrate through fiber digestion, which helps stimulate intestinal nerve activity [42,43]. In addition, by regulating gut barrier function, promoting intestinal motility, and maintaining gut pH levels, it helps prevent constipation and gastrointestinal diseases. Gut microbiota can regulate gut functions through the metabolites of bacterial fermentation, among which 5-hydroxytryptamine (5-HT), SCFAs, methane, and BAs occupied more important positions [44]. Mounting evidence has highlighted the pathological role of gut microbiota disruption in functional constipation, as the findings indicate that functional constipation is linked to a reduction in beneficial species, an increase in pathogenic species, and diminished species diversity in humans. Diverse gut microbiota can regulate the host’s metabolism and immune response by producing SCFAs, vitamins, and other metabolic products.

### 2.2. Mechanisms of Dysbiosis and the Occurrence of Constipation

Dysbiosis refers to an abnormal composition and function of the microbial community in the gut, typically characterized by a decrease in beneficial bacteria such as bifidobacteria and lactobacilli and an increase in harmful bacteria such as *Escherichia coli* and *Klebsiella* [45,46]. This imbalance can be caused by various factors, including poor diet, antibiotic use, infections, chronic diseases, and changes in lifestyle. For constipation patients, research shows that the gut microbiota is significantly less diverse, with fewer beneficial bacteria (e.g., *Firmicutes* and *Bacteroidetes*) and more potentially harmful bacteria (e.g., *Escherichia coli*) [43,47]. Normally, these receptors enhance smooth muscle motility through calcium signaling pathways when stimulated by DCA. Furthermore, the occurrence of constipation is closely related to a decrease in the diversity of gut microbiota; this reduction in diversity may affect the normal functioning of the gut, leading to the onset of constipation [48,49].

**Figure 1:**
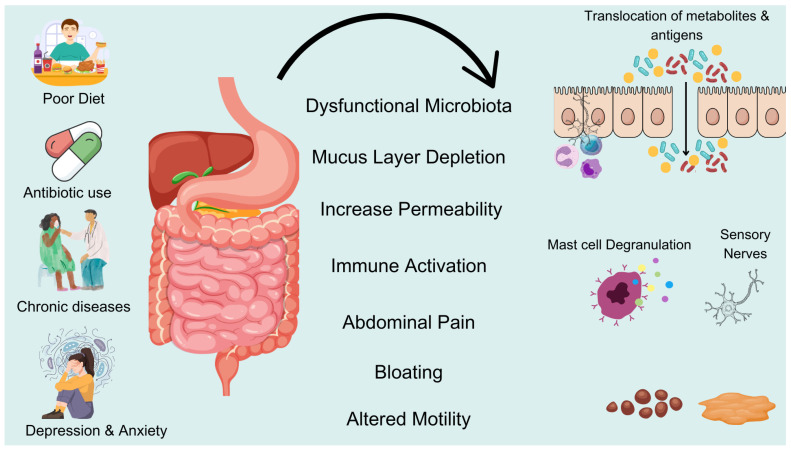
Imbalance in gut flora leads to constipation. Poor diet, antibiotics, chronic diseases, and stress can disrupt gut microbiota, leading to mucus layer depletion and increased intestinal permeability. This dysfunction triggers immune activation and sensory nerve stimulation, causing abdominal pain, bloating, and altered motility. These changes contribute to constipation and other gastrointestinal disorders.

Dysbiosis of the gut microbiota can lead to abnormalities in gut motility, secretion, and immunity by affecting the nerves and motility of the intestine. For example, the increase in harmful bacteria such as the abundance of gut Bacteroidetes may lead to inflammatory responses [50], causing a slowdown in intestinal motility; while the reduction in probiotics can result in insufficient production of short-chain fatty acids, thereby affecting bowel function. Therefore, dysbiosis may become a direct factor in the occurrence of constipation. First, gut microbiota can regulate the function of gut neurons and promote intestinal motility by producing metabolites such as SCFAs [51]. SCFAs can enhance the growth and function of gut neurons, thereby improving intestinal motility. Additionally, dysbiosis may lead to inflammatory responses in the enteric nervous system, which can further affect intestinal motility. Studies have found that, in the intestines of patients with constipation, the absence of certain beneficial bacteria, such as coprococcus, is associated with dysfunction of gut neurons [50], which may be one of the reasons for weakened intestinal motility. In patients with constipation, the increase in harmful bacteria in the gut may stimulate the immune system, leading to the release of inflammatory factors, which in turn exacerbate constipation symptoms [52]. Furthermore, the inflammatory response can further aggravate dysbiosis, creating a vicious cycle. There is a complex interaction between dysbiosis and inflammatory responses. Dysbiosis can lead to impaired gut barrier function and increased intestinal permeability, triggering systemic inflammatory responses, while the improvement of gut microbiota, such as probiotics and fecal microbiota transplantation, can effectively alleviate the inflammatory responses in rat models of constipation and in patients with constipation [53,54], thus improving constipation symptoms. The main mechanisms of dysbiosis and the occurrence of constipation are shown in Figure 1.

### 2.3. Mechanisms by Which the Gut Microbiota Regulates Intestinal Motility and Functions

The enteric nervous system (ENS) operates independently of the central nervous system (CNS) and has autonomous control capabilities, often referred to as the “second brain” [55], there exists a complex signaling network between the ENS and the gut microbiota. The ENS controls most aspects of bowel function [56]. The ENS is a complex network of neurons and glial cells, containing 500 million neurons (approximately the total number of spinal cord neurons) and a network of glial cells. The ENS located in different areas is involved with different functions. The muscle plexus named Auerbach’s plexus, located in the intermuscular nerve plexus between the longitudinal and circular muscles, primarily controls the contraction and relaxation of the muscles. The submucosal intestinal nerve plexus, Meissner’s plexus, situated between the circular muscles and the intestinal mucosa, regulates fluid secretion and absorption, modulates blood flow, and responds to stimuli from the epithelium and lumen to support intestinal function [55]. There is a bidirectional communication between the ENS and the gut microbiota [57]. As shown in Table 1, gut microbiota acts on the ENS through multiple mechanisms of action. Simultaneously, the ENS orchestrates gut microbiota composition through multifaceted regulatory mechanisms. Emerging evidence reveals that rhythmic migrating motor complex (MMC) activity will shape the dynamics of microbial spatial organization along the gastrointestinal tract, creating distinct ecological niches through mechanical clearance patterns [58]. Complementing this neural-motor control, epithelial-derived defensin α6 selectively modulates microbial colonization by exerting charge-dependent bactericidal effects against specific bacterial taxa, thereby restructuring mucosal adherence landscapes [59]. Among the host mediators of intestinal inflammation, NO is an essential component of host immunity. NO plays an important role during infections by limiting microbial proliferation, which can target nucleic acids and lipid components with bactericidal effects [60,61]. NO in vitro fermentation models showed that NO induced both transient and long-term modifications of the gut environment, microbial composition, and metabolism [61].

Emerging evidence reveals that diverse oxidative stress biomarkers beyond nitric oxide modulate gut microbial ecology through distinct mechanisms. Primarily, the excessive generation of reactive oxygen species (ROS) compromises intestinal mucus barrier integrity, impairing *Akkermansia muciniphila* colonization capacity while inducing a *Firmicutes*-to-*Bacteroidetes* ratio imbalance, thereby disrupting SCFA biosynthesis [62]. Subsequently, diminished antioxidant enzyme activities, particularly superoxide dismutase (SOD) and glutathione peroxidase (GPx), potentiate oxidative damage to beneficial microbiota including Bifidobacterium and *Lactobacillus* spp. [63,64]. Concomitantly, the aberrant accumulation of lipid peroxidation end-products, such as malondialdehyde (MDA), stimulates opportunistic pathogen proliferation while suppressing the metabolic functions of butyrate-producing species. Notably, protein carbonyl derivatives drive microbial community restructuring through TLR4/NF-κB pathway activation, favoring proteobacterial dominance. Ultimately, elevated 8-hydroxy-2′-deoxyguanosine (8-OHdG) levels demonstrate significant negative correlation with Lactobacillus abundance, potentially inducing intestinal dysmotility via interference with SCFA metabolic pathways. These interconnected oxidative stress parameters collectively orchestrate microbial population dynamics and metabolic networks, constituting critical pathophysiological determinants of gastrointestinal dysfunction.

In shaping the immune microenvironment, activated type 3 innate lymphoid cells (ILC3) regulate the dynamic balance of Th17/Treg cells through the IL-22/STAT3 signaling axis, creating an immune tolerant environment conducive to the proliferation of symbiotic bacteria [65]. These interconnected ENS-driven processes collectively maintain microbial biogeography and functional homeostasis through neural–epithelial–immune crosstalk, as demonstrated in recent mechanistic studies.

In the field of clinical disease pathophysiology research, the imbalance in the interaction between the enteric nervous system and the microbiota shows significant disease-specific characteristics. Patients with irritable bowel syndrome (IBS) often exhibit a significant reduction in enteric neuron density, accompanied by abnormal expression of the serotonin transporter [66]. Probiotic intervention can increase the expression of transporter proteins by regulating the abundance of butyrate-producing bacteria. Meanwhile, in the research field of inflammatory bowel disease (IBD), clinical cohort analyses have found a significant positive correlation between the serum concentration of the enteric nervous system glial cell-specific marker S100β and fecal calprotectin levels [67], providing a new approach for non-invasive monitoring of the degree of ENS damage.

## 3. Exploring Metabolomics in Understanding Constipation Mechanisms

Metabolomics primarily analyzes small molecular metabolites in organisms through high-throughput technologies to reveal biological processes and disease mechanisms [68]. The main techniques of metabolomics include gas chromatography–mass spectrometry (GC-MS), liquid chromatography–mass spectrometry (LC-MS), and nuclear magnetic resonance (NMR) [69]. Advances in these technologies have led to the widespread application of metabolomics in biomedical research, particularly in exploring disease mechanisms, early diagnosis, discovering biomarkers, and personalized medicine. The causes of constipation are complex, and the composition and dysfunction of gut microbiota may be closely related to the occurrence of constipation. By using metabolomics to detect the characteristics of gut microbiota and specific metabolites in patients with FC, such as assessing the biodiversity of gut microbiota and the abundance of beneficial bacteria like butyrate-producing bacteria, we can gain insights into the pathology of constipation. Additionally, metabolites such as butyrate in serum, along with upstream products related to arginine biosynthesis (such as 2-oxoglutarate, L-glutamate, N-acetylornithine, and L-ornithine), and SCFAs can help understand the etiology of constipation and potential intervention measures. The clinical studies on gut microbiota interventions for constipation are shown in Table 2.

### 3.1. Metabolomic Characteristics of Patients with Constipation

Metabolomic studies have revealed that the metabolite profiles in patients with FC often show significant differences compared to healthy individuals, particularly in the production of specific metabolites.

### 3.2. The Impact of Gut Microbial Metabolites on Host Metabolism

The influence of gut microbes on host metabolism is multifaceted, involving microbial composition, metabolite production, and interaction with host physiological functions. It has been shown that in individuals with obesity, diabetes, or both, there are increases in the proportion of the *Firmicutes* to *Bacteroidetes* ratio compared to healthy individuals [70]. The *Bacteroidetes*/*Firmicutes* ratio enhances the host’s ability to extract energy from food and may promote obesity [71,72]. Gut microbes metabolize dietary components, and the metabolites produced have a significant impact on the host’s metabolic functions. SCFAs, particularly acetate, propionate, and butyrate, can increase intestinal motility, promote gut health, inhibit inflammation, and enhance gut barrier function [73]. SCFAs regulate intestinal movement and defecation reflexes by stimulating intestinal neurons and endocrine cells [73,74]. The abundance of bacteria producing SCFAs in the gut microbiota of patients with constipation is significantly reduced, leading to a decreased ability to generate SCFAs. This may result in insufficient intestinal motility, which could be one of the important reasons for the onset of constipation symptoms in patients due to gut microbiota dysbiosis [75,76].

Gut microbiota also regulate metabolism by influencing the host’s neuroendocrine system. For example, certain gut bacteria are capable of synthesizing neurotransmitters such as gamma-aminobutyric acid (GABA), which has been shown to improve bowel motor function and relieve constipation symptoms [77]. Meanwhile, studies have shown that the reduced abundance of GABA-synthesis-associated bacteria in the gut microbiota of patients with constipation may lead to insufficient levels of GABA, thus affecting normal intestinal motility [78]. In addition, the gut microbiota is also involved in the metabolism of BAs, producing free BAs and secondary bile salts. These metabolic products not only affect the digestion and absorption of lipids but also have a profound impact on intestinal motility, inflammation regulation, and the structure of the gut microbiota [79]. In addition, gut microbiota can also produce other secondary metabolites, such as tyramine and indole, which can interact with the host’s intestinal receptors to influence gut motility and immune responses [80].

### 3.3. Analysis of Potential Biomarkers of Constipation

Recent studies have shown that alterations in gut microbiota and metabolites can provide insights into the pathophysiology of constipation. For instance, a study involving a constipation model in rats utilized 16S rRNA sequencing and NMR-based metabolomics to illustrate the significant changes in the diversity of intestinal microbial communities and the presence of specific metabolites that are associated with constipation [81]. Constipation not only affects locomotor activity and memory but also leads to alterations in metabolic pathways, with 28 fecal metabolites identified as being associated with the condition, and 14 of these metabolites were proposed as potential diagnostic markers for constipation [82]. Constipation and emotional issues have received widespread attention in recent years, and studies have shown that patients with constipation have a higher probability of suffering from psychological issues such as depression and anxiety [83]. Meanwhile, the prevalence of constipation was twice as high if workers had depression/anxiety [84]. The research shows that gut microbiota composition may be associated with a higher incidence of anxiety and depression in patients with FC, thus providing insight into the mechanisms that ameliorate mood disorders in patients with FC [85]. SCFAs produced by gut microbiota are considered important signaling molecules that regulate neurotransmitters, influencing brain health by affecting neuronal function and inflammatory responses [86].

**Table 2:** Clinical Studies on Gut Microbiota Interventions for Constipation.

Population (n)	Intervention	Key Outcomes	Microbiota/Metabolite Changes	Reference
FC patients (n = 60) vs. Healthy (n = 60)	None (observational)	Butyrate (5.2 vs. 12.3 μM) correlated with slower transit (*p* < 0.01)	Roseburia decreased; Bacteroides increased	[11]
Adults with FC (n = 120)	Prebiotic UG1601 (8 weeks)	Stool frequency (3.1 increased to 5.2/week; *p* = 0.003); improved Bristol score	Bifidobacterium increased; fecal butyrate increased by 40%	[53]
FC patients (n = 45)	*L. rhamnosus* LRJ-1 (12 weeks)	Improved bowel movements (*p* = 0.02); reduced bloating	GABA-producing bacteria (Lactobacillus increased 2.5-fold)	[54]
Refractory FC (n = 30)	FMT (single dose)	70% response rate (with increased stool frequency); effects lasted 6 months	Donor-like microbiota; SCFAs (butyrate) increased by 60%	[84]
Healthy adults (n = 40)	Arabinoxylan oligosaccharides	Improved transit time (*p* = 0.04); no adverse effects	Bifidobacterium; propionateincreased by 35%	[83]

Moreover, a comprehensive analysis of fecal and plasma metabolomes in patients with functional gastrointestinal disorders has illustrated that metabolites linked to bile acid and amino acid metabolism were particularly significant, providing a biochemical basis for the observed symptoms [87]. This aligns with findings from another study which reveal specific microbial signatures and metabolic disturbances that could serve as biomarkers for the disorder [88]. In addition, dietary interventions, such as the administration of xylooligosaccharides (XOS), have been shown to modulate gut microbiota and metabolic pathways [89]. Furthermore, the application of machine learning techniques to analyze large datasets from multiple cohorts has led to the identification of key genera, such as Serratia and Dorea, as potential biomarkers for constipation [90]. This approach not only enhances our understanding of the microbiome’s role in constipation but also paves the way for the development of targeted therapeutic strategies based on microbial and metabolic profiles.

The integration of metabolomics and microbiome analysis presents a robust framework for identifying potential biomarkers for constipation, offering new avenues for diagnosis and treatment. The ongoing research in this field underscores the need for further exploration of the complex interplay between gut microbiota, metabolites, and host physiology to develop effective interventions for constipation management.

## 4. Mechanism of Gut Microbiota Metabolites and Constipation

**Figure 2:**
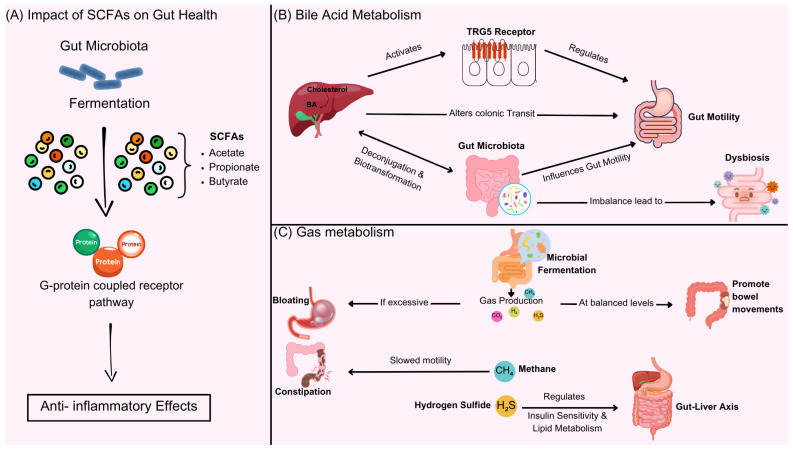
Gut microbiota metabolites, including SCFAs, bile acid derivatives, neurotransmitters, and gas molecules, play a key role in intestinal function. (**A**) SCFAs and gut health—gut microbiota ferment dietary fiber to produce SCFAs, including acetate, propionate, and butyrate, which activate G-protein coupled receptors to exert anti-inflammatory effects and regulate gut function. (**B**) Bile acid metabolism—gut microbiota modulate BAs metabolism through deconjugation and biotransformation, influencing intestinal motility. Bile acids activate FXR/TGR5 receptors, regulating colonic transit. Dysbiosis can disrupt this balance, leading to motility disorders. (**C**) gas metabolism—microbial fermentation produces gases such as hydrogen, methane, and hydrogen sulfide. In balanced amounts, these gases promote bowel movements, but excessive gas or altered composition (e.g., increased methane or hydrogen sulfide) can slow motility, contributing to constipation and metabolic dysregulation via the gut–liver axis.

The metabolic products of gut microbiota mainly include SCFAs, such as acetate, propionate, butyrate, bile acid derivatives, neurotransmitters (such as serotonin), and gas molecules (such as hydrogen). SCFAs improve constipation by regulating the energy metabolism of intestinal epithelial cells, promoting mucus secretion, and enhancing intestinal motility. BAs regulate intestinal fluid secretion and motility rhythms by activating FXR/TGR5 receptors; changes in the metabolite profile caused by gut microbiota imbalance (such as a decrease in hydrogen-producing bacteria and an increase in hydrogen sulfide) may lead to constipation by inhibiting smooth muscle contraction. Research shows that propionate produced by specific bacterial genera (such as *Akkermansia*) can enhance the intestinal barrier and alleviate inflammation-related constipation. The mechanism is shown in Figure 2 and Table 3.

### 4.1. The Impact of SCFAs on Gut Health

SCFAs are important metabolic products generated by gut microbiota through the fermentation of dietary fibers, mainly including acetate, propionate, and butyrate. SCFAs play a crucial role in maintaining gut health and function, and their mechanisms affecting constipation include the stimulation of intestinal motility, anti-inflammatory effects, and intestinal barrier function. SCFAs can enhance intestinal peristalsis by stimulating the smooth muscle of the gut, thereby promoting bowel movements [80]. Butyric acid is considered one of the strongest SCFAs affecting intestinal motility and can directly regulate the intestinal nervous system [91]. SCFAs can inhibit inflammatory responses in the gut, regulate the immune system, reduce the level of inflammation in the intestinal environment, and improve symptoms related to constipation [92]. The deficiency of SCFAs is considered an important biomarker for constipation. Compelling evidence underscores the critical role of SCFAs insufficiency in the pathogenesis of intestinal motility impairments, with multiple cohort studies establishing its strong correlation with constipation severity and duration. By measuring the concentrations of SCFAs in feces and blood, researchers can assess gut function and health status [93]. Elevated levels of certain amino acids (such as tryptophan derivatives) may indicate metabolic disorders in patients with constipation, related to changes in gut microbiota [94]. In terms of obesity and diabetes-related metabolites, studies have also found that some metabolites associated with metabolic syndrome (such as certain ketone bodies and free fatty acids) show level changes in patients with constipation, which may help identify potential links between constipation and metabolic diseases [95].

### 4.2. BAs Metabolism and Intestinal Motility

BAs play a crucial role in the regulation of gut motility, acting as signaling molecules that influence various gastrointestinal functions. The gut microbiota is integral to bile acid metabolism, as it can modify BAs through deconjugation and biotransformation, which in turn affects gut motility. Studies have shown that alterations in BAs profiles can lead to changes in colonic transit times, with specific BAs like lithocholic acid exhibiting pro-motility effects through the activation of the bile acid receptor TGR5 [96]. This receptor is expressed in the enteric nervous system and is involved in mediating the effects of BAs on gut motility, indicating a complex interplay between BAs signaling and gut microbiota composition. Furthermore, dysbiosis, or an imbalance in gut microbiota, can lead to impaired BAs metabolism, contributing to gastrointestinal disorders such as functional constipation [97]. The relationship between gut microbiota, bile acid metabolism, and gut motility suggests that therapeutic strategies aimed at restoring microbial balance could improve gut motility and alleviate symptoms of constipation. In addition, the gut–brain axis highlights how gut microbiota can not only influence local gastrointestinal function but also systemic effects on health. The metabolites produced by gut microbiota, including BAs and SCFAs, can affect neurotransmitter signaling pathways, thereby influencing gut motility and overall gastrointestinal health [77]. Interventions targeting gut microbiota, such as probiotics and dietary modifications, have shown promise in enhancing bile acid metabolism and improving gut motility, offering potential therapeutic avenues for managing gastrointestinal disorders associated with dysbiosis [98].

Overall, the intricate relationship between bile acid metabolism, gut microbiota, and gut motility underscores the importance of maintaining a balanced gut microbiome for optimal gastrointestinal function. Future research should focus on elucidating the specific mechanisms by which gut microbiota influence bile acid metabolism and gut motility, as well as exploring targeted interventions that can restore microbial balance and improve gastrointestinal health.

### 4.3. Gas Metabolism and Regulation of Intestinal Function

The interplay between gas metabolism and gut function is a complex and multifaceted area of research that highlights the significant role of gut microbiota in health and disease. Gas production in the gastrointestinal tract is primarily a result of microbial fermentation of dietary substrates, leading to the generation of gases such as hydrogen, methane, and carbon dioxide. These gases can influence gut motility, mucosal integrity, and even systemic metabolic processes. Recent studies have demonstrated that alterations in gut gas composition are associated with various gastrointestinal disorders, including constipation, inflammatory bowel disease, and obesity. For instance, under normal circumstances, the production of gas in the intestines can promote bowel movements by increasing intraluminal pressure [99], but when gas production is excessive or release is obstructed, it can instead lead to bloating and constipation [100]. Certain gas metabolic products may affect intestinal permeability and mucosal health, thereby impacting overall intestinal function [101]. At the same time, changes in gas types may reflect the composition of gut microbiota, as a higher presence of methane-producing microorganisms may be associated with a tendency towards constipation [102]. Methane can slow down intestinal motility, thereby triggering symptoms of constipation.

Furthermore, the regulation of gut function through gas metabolism is not limited to local effects. Emerging evidence suggests that gut-derived gases can influence systemic metabolic pathways, including insulin sensitivity and lipid metabolism. For example, hydrogen sulfide (H_2_S), a gas produced by specific gut bacteria, has been shown to have protective effects on insulin secretion and sensitivity [103]. This underscores the importance of understanding the gut–liver axis and how microbial metabolites, including gases, can modulate host metabolism and contribute to the pathogenesis of metabolic disorders. Additionally, the therapeutic potential of modulating gas production through dietary interventions or probiotics presents a promising avenue for managing gastrointestinal disorders and improving overall gut health.

Overall, the intricate relationship between gas metabolism and gut function underscores the need for further research to elucidate the underlying mechanisms and potential therapeutic strategies for gastrointestinal disorders.

## 5. The Potential Applications of Metabolomics in Personalized Treatment of Constipation

Metabolomics has established multidimensional avenues for personalized constipation management. In diagnostics, metabolic signatures (e.g., butyrate deficiency, tryptophan metabolism dysregulation) identified through GC-MS/LC-MS platforms enable precise subtype classification and early screening. Therapeutically, monitoring metabolite changes (particularly SCFAs) following probiotic interventions (e.g., Bifidobacterium), dietary fiber supplementation, and fecal microbiota transplantation guides optimized microbiota-targeted regimens. Future research must address three critical challenges establishing standardized analytical protocols, implementing longitudinal multiomics integration (genome–-metabolome associations), and resolving ethical/accessibility barriers in clinical translation. These advancements collectively drive constipation management toward a closed-loop model of “metabolic signature guidance–precise microbiota modulation–dynamic outcome monitoring”.

### 5.1. Innovative Approaches in Personalized Diagnosis of Constipation: Metabolomics-Driven Strategies

Metabolomics has introduced novel perspectives for developing individualized diagnostic frameworks for constipation. Contemporary research focuses on deciphering patient-specific metabolic profiles to identify clinically relevant biomarkers. Current metabolomics-based diagnostic strategies encompass three principal components: targeted biomarker discovery, metabolic signature mapping, and integrative omics analysis. By using advanced analytical platforms, including GC-MS and LC-MS, we enable a systematic comparison of biofluids (serum, urine) and fecal samples between constipation patients and healthy controls. Multivariate analysis has revealed diagnostic signatures such as depleted butyrate levels (indicative of impaired SCFAs metabolism) and dysregulated tryptophan catabolism pathways. These biomarkers not only facilitate early detection but also the etiological stratification of constipation subtypes.

Beyond conventional diagnostic criteria, metabolic fingerprinting techniques have demonstrated clinical utility in differentiating functional constipation from irritable bowel syndrome [82]. We may have hope to identify genetic variations related to constipation and their corresponding metabolic changes through the joint analysis of genomics and metabolomics, providing a theoretical basis for precision medicine and promoting the development of personalized treatment strategies.

### 5.2. Metabolic Strategies for Treating Constipation by Regulating Gut Microbiota

Regulating the gut microbiota is considered an effective strategy for improving constipation, and metabolomics can provide important data support in this field. The gut microbiota can be adjusted through probiotic interventions, dietary interventions, and the increasingly popular clinical practice of fecal microbiota transplantation (FMT) in recent years. Studies have shown that specific probiotics (such as *Bifidobacteria* and *Lactobacilli*) can improve the gut microbiota composition in patients with constipation and increase the production of SCFAs [104,105], thereby promoting intestinal motility. Metabolomics allows us to systematically assess metabolic changes after probiotic interventions, helping to determine the optimal probiotic combinations. High-fiber diets have been proven to improve constipation, and metabolomics can be used to analyze the effects of different dietary components on changes in gut microbiota and their metabolic products (such as SCFAs) [106]. By systematically analyzing the metabolic responses of the human body under different dietary interventions, personalized nutritional intervention plans can be developed. Furthermore, metabolomics can monitor metabolic changes after FMT, such as changes in SCFAs and amino acids, providing objective biomarkers for the therapeutic effects of constipation, a method that has shown potential in several clinical studies [107]. Emerging therapies combine probiotics (such as *Lactobacillus rhamnosus* GG) with antioxidants (N-acetylcysteine, glutathione) to mitigate oxidative stress. For example, NAC supplementation reduces ROS by 40% and restores *Faecalibacterium abundance*, improving motility in refractory constipation [63].

### 5.3. Future Research Directions and Challenges

The exploration of the gut microbiome and its interactions with host metabolism is a rapidly evolving field that presents numerous research opportunities and challenges. Recent studies have highlighted the complex relationships between gut microbiota composition, metabolomic characteristics, and the pathophysiology of diseases such as functional constipation and irritable bowel syndrome. Existing metabolomic studies lack standardized protocols for sample processing and data analysis, necessitating the establishment of standardized research designs and analytical processes to ensure the reproducibility and reliability of research findings [108,109]. Secondly, most current research focuses on the cross-sectional changes in metabolites, and future studies should conduct longitudinal research to observe the changes in metabolic characteristics of patients with constipation and the durability of treatment effects [110]. Clinical practice should strengthen the integration of metabolomics with other omics data (such as genomics, transcriptomics, and proteomics) to form a systems biology perspective, which will help to gain a more comprehensive understanding of the mechanisms of constipation and provide stronger data support for personalized treatment. However, the complexity of these interactions requires robust bioinformatics tools and methods to analyze the large amounts of data generated, which remains a barrier to progress. There is a need to enhance communication and collaboration between basic research and clinical applications.

Finally, addressing the ethical considerations and accessibility of microbiome research is crucial. As research increasingly involves diverse populations, ensuring equitable access to interventions and maintaining ethical standards in research practices becomes essential. This includes considering the impact of microbiome research on public health policy and the potential disparities in health outcomes based on socioeconomic factors. Overall, while the future of microbiome research is promising, it is necessary to tackle the associated challenges to translate research findings into effective clinical applications.

## 6. Concluding Perspectives

The synergistic integration of metabolomics with gut microbiota research is poised to fundamentally transform our approach to constipation management. While current studies have successfully mapped over 120 constipation-associated metabolites and identified 18 microbial taxa with prokinetic potential, three critical gaps demand urgent attention. Firstly, the lack of longitudinal datasets tracking metabolic–-microbial dynamics during therapeutic interventions. Secondly, the insufficient resolution of spatial metabolome variations across intestinal subregions. Thirdly, ethical concerns regarding personalized microbiome modulation. Future research must prioritize the development of AI-powered metabolic flux analyzers capable of real-time tracking microbial metabolite production, coupled with organoid-based validation platforms that bridge in vitro findings to human pathophysiology.

Emerging technologies such as wearable colonic sensors and CRISPR-engineered microbial consortia present unprecedented opportunities for targeted interventions. However, their clinical implementation requires rigorous standardization through international consortia like the Global Metabolomic Initiative for Gastrointestinal Health (GMIGH). We propose establishing metabolomic “reference human” baselines stratified by ethnicity, diet patterns, and microbiome enterotypes to enable meaningful cross-population comparisons.

The ultimate breakthrough may lie in decoding the gut–brain metabolic dialog, particularly, the microbial synthesis of neuroactive metabolites (e.g., tryptamine derivatives) that modulate enteric nervous system activity. This paradigm shift from stool pattern observation to molecular pathway manipulation could redefine functional constipation as a treatable metabolic disorder rather than a symptomatic diagnosis. Through coordinated efforts across metabolomics, synthetic biology, and neurogastroenterology, we stand at the threshold of developing precision microbial therapeutics that restore intestinal homeostasis at its metabolic roots.

## Figures and Tables

**Table 1:** Gut microbiota-ENS Regulatory Pathway.

Mode of Action	Key Mediators	Molecular Mechanisms	Functional Impacts
Metabolite signaling	SCFAs (butyric acid/propionic acid)	Activate the FFAR2/3 receptor of ENS neurons	Enhances intestinal contraction frequency (+35%)
Neurotransmitter precursor supply	Tryptophan → 5-HT	Activation of TPH1 enzyme in enterochromaffin cells	Modulates peristaltic rhythm
Immune-mediated regulation	IL-6/IL-1β	Activation of TLR4/NF-K B pathway in glial cells	Induces ENS hypersensitivity
Epigenetic modifications	Histone deacetylase inhibition	Inhibition of HDAC2/3 activity in ENS neurons	Promotes neuroplasticity-associated gene expression

**Table 3:** Expanded Integration of Metabolomic, Microbial, and Clinical Biomarkers in Chronic Constipation.

Target	Change in FC	Levels in FC vs. Healthy	Function	Clinical Correlation	Therapeutic Response	Detection Method	Reference
Butyrate	50–60% decreased	5.2 vs. 12.3 μM (fecal)	Energy for colonocytes, motility	Correlates with stool frequency (r = 0.58)	Probiotics levels increased by 40%	GC-MS	[51]
Propionate	40% decreased	8.1 vs. 13.5 μM (fecal)	Anti-inflammatory, barrier	Links to visceral hypersensitivity	Prebiotics restore in 8 weeks	LC-MS	[82]
Acetate	35% decreased	15.2 vs. 23.4 μM (fecal)	pH regulation, mucus production	Associated with hard stools (*p* < 0.05)	FMT normalizes in 4 weeks	NMR	[83]
Deoxycholic acid (DCA)	40% dercreased	1.1 vs. 1.8 mM (fecal)	TGR5 activation → motility	Predicts slow transit (AUC = 0.71)	Rifaximin increased 2-fold	LC-MS	[72]
Lithocholic acid (LCA)	30% decreased	0.8 vs. 1.2 mM (fecal)	Anti-inflammatory	Correlates with bloating severity	Ursodeoxycholic acid improves	LC-MS/MS	[73]
Tryptophan	30% decreased	5.9 vs. 8.4 μg/mL (serum)	Serotonin precursor	Links to depression (OR = 2.1)	Probiotics restore to 90% normal	HPLC	[54]
GABA	45% decreased	2.1 vs. 3.8 μg/mL (serum)	ENS modulation	Correlates with anxiety (r = 0.42)	*L. rhamnosus* increased 2.5-fold	ELISA	[63]
*Roseburia* spp.	70% decreased	3.1% vs. 10.4% (rel. abundance)	Butyrate production	Predicts fiber response (*p* = 0.02)	XOS abundance increased 2.5-fold	16S rRNA	[66]
*Akkermansia muciniphila*	80% decreased	0.5% vs. 2.5% (rel. abundance)	Mucus layer maintenance	Links to IBS overlap (*p* = 0.03)	*L. plantarum* increased 60%	qPCR	[82]
*Bifidobacterium* spp.	50% decreased	4.2% vs. 8.4% (rel. abundance)	Acetate production	Correlates with stool consistency	Probiotic cocktails increased 3-fold	Metagenomics	[81]
Methane (CH_4_)	2-fold increased	≥3 ppm vs. ≤1 ppm (breath)	Slows motility	OR = 3.2 for bloating	Low-FODMAP diet decreased 50%	Breath test	[79]
Hydrogen sulfide (H_2_S)	3-fold increased	2.4 vs. 0.8 mM (fecal)	Inhibits smooth muscle	Links to pain severity (VAS increased 20%)	Bismuth subsalicylate reduces	GC	[78]
Butyrate + Tryptophan	Combined decreased	N/A	FC vs. IBS-C differentiation	Sensitivity 85%, Specificity 78%	Probiotics improve panel accuracy	LC-MS/NMR	[81]
2-Oxoglutarate	60% decreased	0.4 vs. 1.0 μM (serum)	Arginine metabolism	Predicts fiber response (*p* = 0.01)	Dietary arginine supplementation	GC-MS	[75]

## Data Availability

No new data were created or analyzed in this study.

## Abbreviations

The following abbreviations are used in this manuscript:

VTE	Venous thromboembolism
BAs	Bile acids
SCFAs	Short-chain fatty acids
FC	Functional constipation
DCA	Deoxycholic acid
ENS	Enteric nervous system
CNS	Central nervous system
MMC	Migrating motor complex
NO	Nitric oxide
IBS	Irritable bowel syndrome
IBD	Inflammatory bowel disease
GC-MS	Gas chromatography–mass spectrometry
LC-MS	Liquid chromatography–mass spectrometry
NMR	nuclear magnetic resonance
GABA	Gamma-aminobutyric acid
XOS	Xylooligosaccharides
FMT	Fecal microbiota transplantation
GMIGH	Global Metabolomic Initiative for Gastrointestinal Health
